# Identification of PARN nuclease activity inhibitors by computational-based docking and high-throughput screening

**DOI:** 10.1038/s41598-023-32039-z

**Published:** 2023-03-31

**Authors:** Thao Ngoc Huynh, Siddharth Shukla, Philip Reigan, Roy Parker

**Affiliations:** 1 Department of Biochemistry, University of Colorado Boulder, Boulder, CO 80303 USA; 2 Howard Hughes Medical Institute, Chevy Chase, MD 20815 USA; 3 Department of Pharmaceutical Sciences, Skaggs School of Pharmacy and Pharmaceutical Sciences, University of Colorado Anschutz, Aurora, CO 80045 USA

**Keywords:** Drug discovery, Molecular biology

## Abstract

Poly(A)-specific ribonuclease (PARN) is a 3′-exoribonuclease that removes poly(A) tails from the 3′ end of RNAs. PARN is known to deadenylate some ncRNAs, including hTR, Y RNAs, and some miRNAs and thereby enhance their stability by limiting the access of 3′ to 5′ exonucleases recruited by oligo(A) tails. Several PARN-regulated miRNAs target p53 mRNA, and PARN knockdown leads to an increase of p53 protein levels in human cells. Thus, PARN inhibitors might be used to induce p53 levels in some human tumors and act as a therapeutic strategy to treat cancers caused by repressed p53 protein. Herein, we used computational-based molecular docking and high-throughput screening (HTS) to identify small molecule inhibitors of PARN. Validation with in vitro and cell-based assays, identified 4 compounds, including 3 novel compounds and pyrimidopyrimidin-2-one GNF-7, previously shown to be a Bcr-Abl inhibitor, as PARN inhibitors. These inhibitors can be used as tool compounds and as lead compounds for the development of improved PARN inhibitors.

## Introduction

Poly(A)-specific ribonuclease (PARN) is a 3′ to 5′ exonuclease that removes poly(A) or oligo(A) tails from the 3′ ends of RNAs^1–4^. PARN is expressed ubiquitously in almost all tissues of eukaryotic organisms^5^ and has multiple functions in eukaryotes. For example, during early development PARN plays a role in mRNA deadenylation in Xenopus^5–7^.

**Figure 1:**
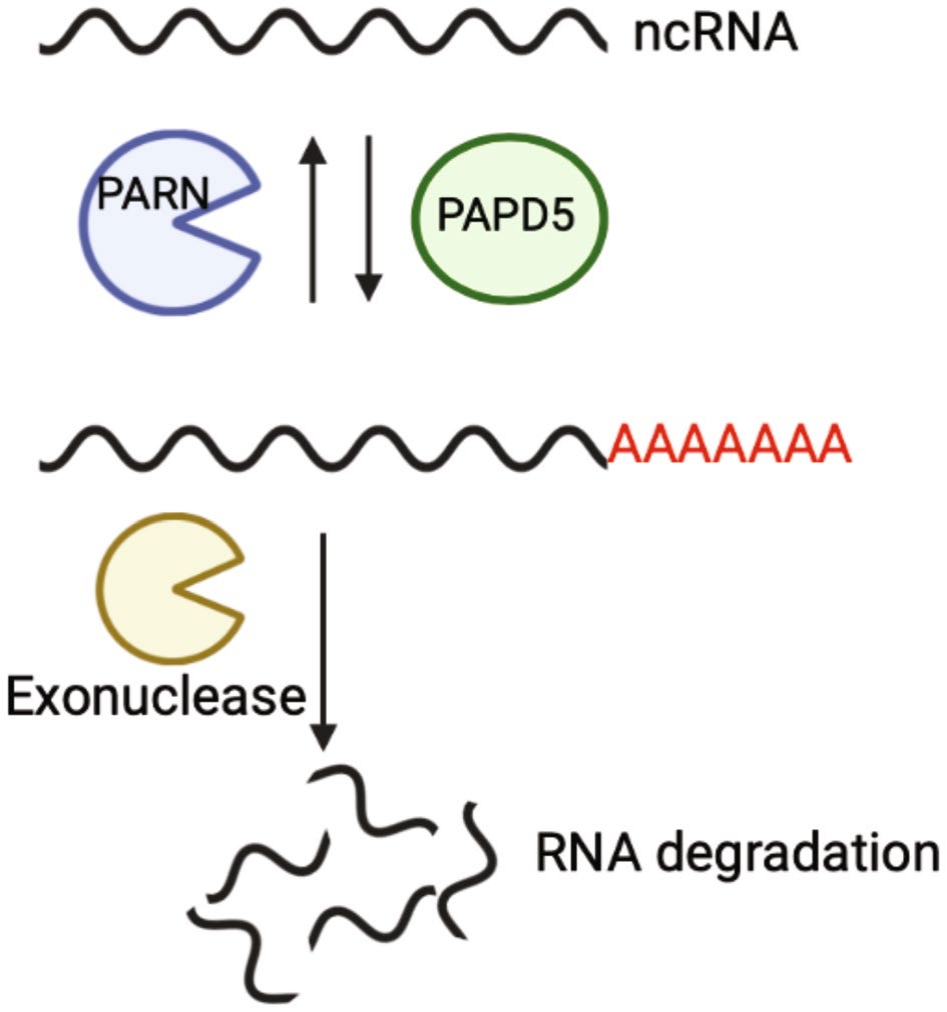
PARN functions in an adenylation/deadenylation regulatory pathway that regulates the decay rate of ncRNAs. ncRNAs could be targeted to adenylation by PAPD5 or deadenylation by PARN^8^. In PARN-deficient cells, the presence of oligo(A) tail can recruit 3′ to 5′ exonucleases to degrade ncRNAs.

In human cells, PARN primarily functions in an adenylation/deadenylation regulatory pathway that regulates the decay rate of ncRNAs (Fig. 1)^8^. In this pathway, Y RNAs, snoRNAs, the human telomerase RNA (hTR), and some miRNAs can be oligoadenylated by noncanonical poly(A) polymerases, such as paralogs PAPD5 and PAPD7^8–12^. The presence of the oligo(A) tail can then recruit processive sequence-independent 3′ to 5′ exonucleases to degrade ncRNAs^9,11,13–17^. Alternatively, the oligoadenylated tail can be removed by adenosine specific 3′ to 5′ exonucleases such as PARN to maintain stability of ncRNAs. Thus, when PARN is inhibited or defective, some ncRNAs are prematurely degraded, including hTR^9–12,17–20^.

PARN also stabilizes some miRNAs by removing poly(A) tails added by PAPD5, which prevents the recruitment of exonucleases DIS3L or DIS3L2 to degrade miRNAs^9,11^. Importantly, several PARN-regulated miRNAs (miR-380-5p, miR-1285, miR-92, miR-214, miR-485, miR-331, miR-665, miR-3126, and miR-25) either have been shown, or are predicted, to target the TP53 mRNA^21–25^. p53 is tumor suppressor that prevents outgrowth of aberrant cells by inducing cell-cycle arrest, DNA repair or programmed cell death^26^. It has been shown that numerous human cancers increase proliferation and resistance to DNA-damage agents by downregulating the p53 pathway^27,28^. Moreover, depletion of PARN upregulates p53 and sensitizes cells to chemotherapeutic agents^11,29^. Thus, inhibition of PARN might be an effective intervention to induce the expression of p53 in some tumors and thereby limit tumor progression.

Currently, only a limited number of PARN inhibitors exist^30–34^. To identify potential inhibitors of PARN, we performed computational-based docking between human PARN and a small molecule library of adenosine analogs and performed high-throughput screening (HTS) of a small molecule library. The combination of these two approaches allowed us to identify four compounds that inhibit PARN in vitro and also repress PARN activity in Hela cells.

## Results

### Purification of PARN for in vitro assay

**Figure 2:**
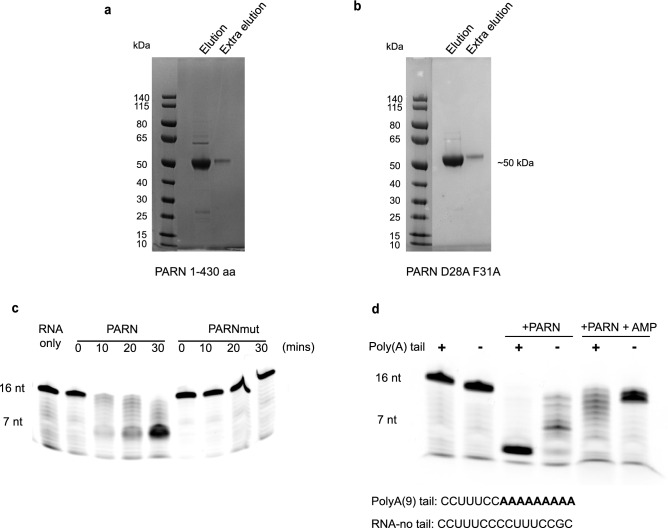
PARN purification and validation. (**a**) SDS-PAGE gel of the fractions during PARN_1-430_ purification. (**b**) SDS-PAGE gel of the fractions during PARNmut purification. (**c**) Gel assay showing PARN_1-430_ and PARNmut’s activity activity on poly(A) tail RNA at different time points (0-, 10-, 20-, and 30-min post incubation), PARNmut shows no enzymatic activity on poly(A) RNA. (**d**) Representative gel confirming PARN activity on poly(A) RNA and non-poly(A) RNA after 20 min of incubation. Poly(A) tail RNA sequence is a fluorescently labeled RNA with a CCUUUCC followed by a 9 nucleotides oligo(A) tail. Non-poly(a) RNA is a fluorescently labeled RNA with a CCUUUCCGC tail instead of 9 nucleotides oligo(A) tail. Full gels are presented in Supplementary Fig. S1.

To test the effects of compounds on PARN, we purified the enzyme and developed an in vitro assay for PARN activity. Expression of full-length PARN led to aggregation, but expression of the C-terminal truncated protein (1-430 aa of PARN) was soluble. Previous work has shown that the D28A and F31A mutations in PARN inhibit PARN activity^35^. Given this, we expressed and purified the catalytic mutant PARN D28A F31A (PARNmut) as a negative control. Purification (see “Materials and methods”) yielded a dominant band for PARN_1-430_ and PARNmut on SDS-PAGE gels (Fig. 2a and b).

We found that purified PARN_1-430_ shows enzymatic activity on the poly(A) tail of test substrates. PARN_1-430_ was incubated with a fluorescently labeled RNA with a CCUUUCC sequence followed by a 9 nucleotide-long poly(A) tail and the reaction product was visualized on denaturing acrylamide gels. We observed that PARN removed the 3′ oligo(A) tail from the RNA substrate (Fig. 2c and d). The activity was dependent on PARN since PARNmut protein showed no removal of the 3′ adenosines (Fig. 2c). Moreover, we observed that PARN is inhibited when treated with adenosine monophosphate (AMP), as has been reported previously^31^. PARN_1-430_ showed reduced activity when incubated with an unadenylated RNA (Fig. 2d), consistent with the finding that PARN_1-430_ preferentially degrades poly(A) tail^36^.

### Developing a high-throughput PARN inhibition assay

**Figure 3:**
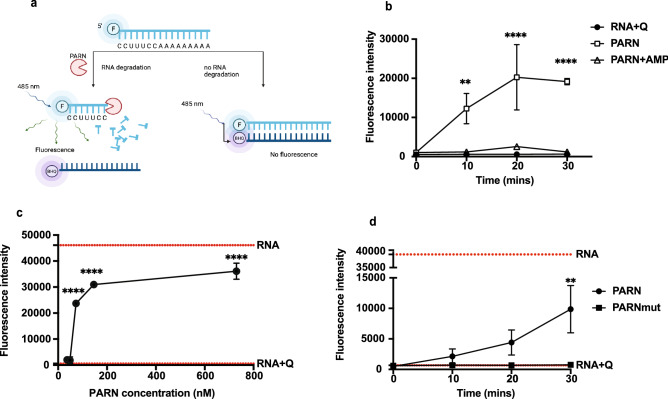
Developing a fluorescence assay for PARN inhibition. (**a**) Cartoon depicting the inhibition fluorescence assay. Schematic diagram of the fluorescence-based deadenylase assay. The assay is based on a 5′ FAM-labelled RNA oligonucleotide substrate. After incubation of the substrate in the presence of PARN_1-430_, the reaction is stopped and a 3′ BHQ-labelled DNA oligonucleotide probe complementary to the RNA substrate is added. The fluorescence of intact substrate is quenched upon probe hybridization because of the proximity of the BHQ fluorophore. In contrast, the BHQ-labelled probe cannot hybridize to the FAM-labelled reaction product allowing detection of FAM fluorescence^37^. (**b**) PARN_1-430_ inhibition assay with fluorescence as a readout with time course for PARN_1-430_ treatment (73 nM), PARN_1-430_ inhibition with 2.5 mM AMP, and no-enzyme control. 2-way ANOVA, multiple comparisons test, average ± SD, n = 3 replicates. *P < 0.05, **P < 0.005, ***P < 0.001, ****P < 0.0001, n.s. was not indicated. (**c**) Dose–response curve of PARN_1-430_ enzymatic activity using different PARN_1-430_ concentrations (36.5–730 nM). Dotted-line represents RNA and RNA + Quencher data. One-way ANOVA, multiple comparisons test, average ± SD, n ≥ 3 replicates. *P < 0.05, **P < 0.005, ***P < 0.001, ****P < 0.0001, n.s. was not indicated. (**d**) PARN_1-430_ and PARNmut enzymatic activity measured at different time points. PARN showed an increase in activity versus time while PARNmut showed no activity up to 30 min incubation. Dotted-line represents RNA and RNA + Quencher data. 2-way ANOVA, multiple comparisons test, average ± SD, n = 3 replicates. *P < 0.05, **P < 0.005, ***P < 0.001, ****P < 0.0001, n.s. was not indicated.

To easily screen compounds for inhibition of PARN activity, we developed an assay in which fluorescence was used as the readout for monitoring PARN_1-430_’s activity (Fig. 3a). This assay was modeled on a similar assay developed for Caf1/CNOT7 deadenylase^37^. In this assay, PARN_1-430_ was incubated with a 5′-fluorescently labeled oligoadenylated RNA. In the absence of deadenylation, this substrate RNA can effectively hybridize to a complementary DNA oligonucleotide with a quencher on its 3′ end leading to a loss of fluorescence (Fig. 3a). When the RNA substrate is deadenylated, the quenching oligonucleotide is no longer able to stably hybridize leading to an increase in fluorescence in the reaction.

This assay has several features that make it useful for assessing PARN activity. First, it is time-dependent (Fig. 3b and d). Second, it is dependent on PARN concentration (Fig. 3c). Third, we observed that AMP, which is a product of PARN activity and can inhibit PARN_1-430_ at high concentrations (> 1 mM), effectively inhibited PARN_1-430_ (Fig. 3b)^31^. Finally, we observed that fluorescence correlates with shortening of the 3′ oligo A tail on the substrate by running the material on a gel and observing shortening of the substrate with PARN_1-430_, but no shortening with PARNmut (Figs. 2c and 3d). In this assay, PARN_1-430_ inhibition is inversely proportional to the reaction fluorescence measured as output, which we can use to test possible PARN inhibitors.

### Computational-based library docking to identify potential PARN inhibitors

**Figure 4:**
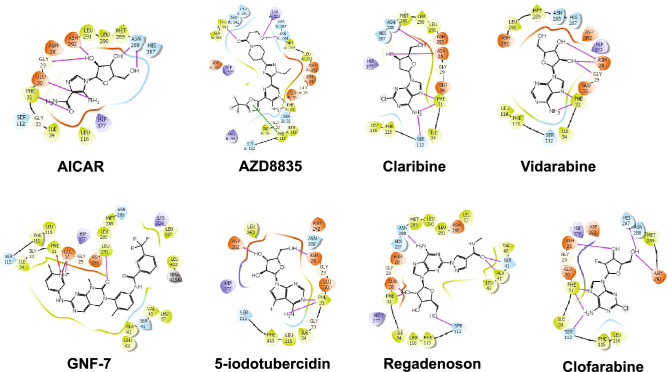
Docking of small molecule adenosine analogs into the PARN catalytic site. Ligand interaction map of the predicted binding mode of AICAR, AZD8835, Claribine, Vidarabine, GNF-7, 5-Iodotubercidin, Regadenson, and Clofarabine, where red residues are charged negative, purple residues are charged positive, green residues are hydrophobic, and blue residues are polar, magenta arrows indicate H-bonds, violet lines indicate salt bridges, and gray spheres represent areas of solvent exposure. HIP represents the ND1 and NE2 protonation state of His and NMA represents *N*-methyl amide of a capped termini. At least one H-bond interaction was observed between the docked small molecule and amino acid residues Asp28-Phe31.

To identify potential small molecule PARN inhibitors, we first used a computational-based docking approach to screen a library of 1820 adenosine analogs from the SelleckChem kinase inhibitor library against the crystal structure of the PARN nuclease domain (PDB: 2A1R)^35^. This library was utilized as the kinase inhibitors are ATP-mimetics and the PARN active site binds adenosine. The PARN nuclease domain includes the four conserved residues among DEDD superfamily, Asp28, Glu30, Asp292, and Asp382, that are important for the catalytic activity of PARN and are required for the binding of divalent metal ions^38^. Mutations of these residues lead to loss of function in PARN^38–40^. Therefore, based on this information, we targeted this catalytic site of PARN and selected high-ranking compounds by XP GScore, an approximation of ligand binding free energy, and by interaction with the Asp28-Phe31 region. Analysis of the docking simulation identified several structurally distinct compounds predicted to dock into the PARN catalytic pocket (Fig. 4).

### Testing compounds predicted to interact with PARN

**Figure 5:**
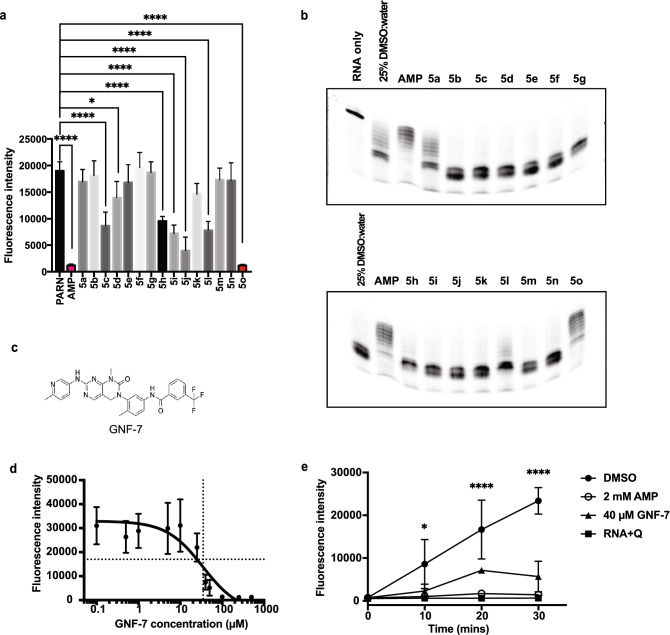
Testing drugs on activity of PARN using RNA substrates in vitro. (**a**) Inhibition fluorescence assay showing effects of different drugs on PARN_1-430_. PARN_1-430_ was pretreated with drugs at room temperature for 10 min before adding RNA substrate. AMP and GNF-7 were shown in pink and red, respectively. One-way ANOVA, multiple comparisons test, average ± SD, n ≥ 3 replicates. *P < 0.05, **P < 0.005, ***P < 0.001, ****P < 0.0001, n.s. was not indicated. (**b**) Gels illustrating inhibitory effects of different drugs on PARN_1-430_. The reaction was performed the same as the fluorescence assay, heat inactivated, then loaded and visualized on gels. Full gels are presented in Supplementary Fig. S1. (**c**) Molecular structure of GNF-7. (**d**) Dose–response curve of GNF-7 on PARN_1-430_. PARN_1-430_ was pre-treated with different concentrations of GNF-7 for 10 min and incubated with RNA substrate. The reaction was quenched with DNA quencher and fluorescence intensity was measured. The IC50 was determined to be 35 ± 15 μM. The vertical dotted line marks the fitted IC50 of GNF-7 and the horizonal dotted line marks 50% inhibition. (**e**) Kinetic analysis of AMP and GNF-7 effects on PARN_1-430_ and a no-enzyme control. Pretreated PARN_1-430_ was incubated with RNA substrate for 0, 10, 20, and 30 min and the fluorescence intensity were measured at each time point. 2-way ANOVA, multiple comparisons, average ± SD, n = 3 replicates. *P < 0.05, **P < 0.005, ***P < 0.001, ****P < 0.0001, n.s. was not indicated.

To determine if any of the compounds predicted to dock to PARN showed effects on PARN_1-430_, we tested 15 compounds based on their docking ranks and commercial availability using the fluorescence assay and gels (Fig. 5 and Table 1). This screen identified 7 compounds that showed PARN_1-430_ inhibition. GNF-7 (labeled as 5o) has the strongest inhibitory effect on PARN_1-430_ (Fig. 5a). In agreement with the fluorescence assay results, the gel assay also revealed GNF-7 (Fig. 5c), a Bcr-Abl inhibitor^41^, as the most effective PARN_1-430_ inhibitor (Fig. 5b). GNF-7 inhibits PARN_1-430_ with a lower concentration compared to AMP (2.5 mM) (Fig. 5a and b).

**Table 1:** List of all the compounds tested as PARN inhibitors with their corresponding properties^30,32–34^.

	Compound name	Rename	PARN inhibitor in vitro	IC50 (µM)	Ki (µM)	PARN inhibitor in cells
Computational docking	5-ITU	5a	No	n.d.	n.d.	n.d.
	5-bromotubercidine	5b	No	n.d.	n.d.	n.d.
	Tubercidine	5c	Yes	n.d.	n.d.	n.d.
	Vidarabine	5d	No	n.d.	n.d.	n.d.
	AZD8835	5e	No	n.d.	n.d.	n.d.
	Clofarabine	5f	No	n.d.	n.d.	n.d.
	AICAR	5g	No	n.d.	n.d.	n.d.
	Spongosine	5h	Yes	n.d.	n.d.	n.d.
	Regadenoson	5i	Yes	n.d.	n.d.	n.d.
	2,6-diamino adenosine	5j	Yes	n.d.	n.d.	n.d.
	2-amino adenosine	5k	No	n.d.	n.d.	n.d.
	Cladribine	5l	Yes	n.d.	n.d.	n.d.
	TWS119	5m	No	n.d.	n.d.	n.d.
	L-adenosine	5n	No	n.d.	n.d.	n.d.
	GNF-7	5o	Yes	34.56	n.d.	Yes
High-throughput screening	SYG-00454609	TH1	Yes	1.39	n.d.	No
	SYG-00457029	TH2	Yes	7.22	n.d.	No
	SYG-00456810	TH3	Yes	5.25	n.d.	No
	SYG-00466189	TH4	Yes	5.11	n.d.	No
	SYG-00465471	TH5	Yes	2.91	n.d.	No
	SYG-00457986	TH6	Yes	8.09	n.d.	No
	SYG-00449761	TH7	Yes	1.64	n.d.	No
	SYG-00446344	TH8	Yes	3.94	n.d.	No
	SYG-00466277	TH9	Yes	5.50	n.d.	No
	SYG-00458754	TH10	Yes	5.71	n.d.	No
	SYG-00457386	TH11	Yes	3.36	n.d.	Yes
	SYG-00459052	TH12	Yes	3.30	n.d.	No
	SYG-00463654	TH13	Yes	6.26	n.d.	No
	SYG-00449350	TH14	Yes	2.50	n.d.	No
	SYG-00456208	TH15	Yes	2.00	n.d.	Yes
	SYG-00445034	TH16	Yes	7.90	n.d.	Yes
	SYG-00462261	TH17	Yes	9.46	n.d.	No
	SYG-00447413	TH18	Yes	0.20	n.d.	No
Ref.^30^	9-(3′,4′, dideoxy-3′-fluoro-β-d-glucopyranosyl)-*N*6-benzoyl adenine	A2	Yes	n.d.	510 ± 52	n.d.
	1-(3′,4′, dideoxy-3′-fluoro-β-d-glucopyranosyl)-*N*4-benzoyl adenine	A6	Yes	n.d.	210 ± 45	n.d.
	3-deoxy-3-fluoro-glucopyranose	B6	Yes	n.d.	n.d.	n.d.
		C6	Yes	n.d.	645 ± 37	n.d.
Ref.^32^	1-(3′-deoxy-3′-fluoro-β-d-glucopyranosyl) uracil	U1	Yes	n.d.	19 ± 5	n.d.
	1-(3′-deoxy-3′-fluoro-β-d-glucopyranosyl) 5-fluorouracil	FU1	Yes	n.d.	98 ± 12	n.d.
	1-(3′-deoxy-3′-fluoro-β-d-glucopyranosyl) thymine	T1	Yes	n.d.	135 ± 18	n.d.
Ref.^33^	Neomycin B		Yes	n.d.	0.4 ± 0.1	n.d.
	Paromomycin		Yes	n.d.	17.3 ± 3.5	n.d.
	Lividomycin		Yes	n.d.	18.7 ± 2.8	n.d.
	Kanamycin B		Yes	n.d.	7.3 ± 0.4	n.d.
	Kanamycin A		Yes	n.d.	64.7 ± 7.8	n.d.
	Tobramycin		Yes	n.d.	7.1 ± 0.2	n.d.
Ref.^34^		5a	Yes	84.1 ± 6.7	n.d.	n.d.
		5b	Yes	n.d.	n.d.	n.d.
		5c	Yes	119 ± 25	n.d.	n.d.
		5d	Yes	125 ± 32	n.d.	n.d.
		5e	Yes	245 ± 20	n.d.	n.d.
		5f	Yes	n.d.	n.d.	n.d.
		5g	Yes	n.d.	n.d.	n.d.
		5h	Yes	n.d.	n.d.	n.d.
		5i	Yes	n.d.	n.d.	n.d.
		5j	Yes	n.d.	n.d.	n.d.
		5k	Yes	n.d.	n.d.	n.d.
		8a	Yes	n.d.	n.d.	n.d.
		8b	Yes	n.d.	n.d.	n.d.
		8d	Yes	n.d.	n.d.	n.d.
		8e	Yes	n.d.	n.d.	n.d.
		8f	Yes	n.d.	n.d.	n.d.
		8j	Yes	23.9 ± 3.7	n.d.	n.d.
		8k	Yes	n.d.	n.d.	n.d.

A dose response curve and kinetic analysis demonstrated that GNF-7 inhibits PARN_1-430_ in a dose-dependent manner (Fig. 5d). The IC50 of GNF-7 on PARN_1-430_ was determined by non-linear fit to be 35 $\pm$ 13 μM. This identified GNF-7 as a potential inhibitor of PARN_1-430_ based on in vitro analyses.

### High throughput screening of Enamine kinase library

To identify PARN inhibitors in a high-throughput manner, 24,000 compounds from the Enamine kinase library were tested in a HTS utilizing the fluorescence assay (Fig. 3a). The top 18 compounds with IC50s of less than 10 μM based on testing at different concentrations (Table 1) were then selected for further testing.

**Figure 6:**
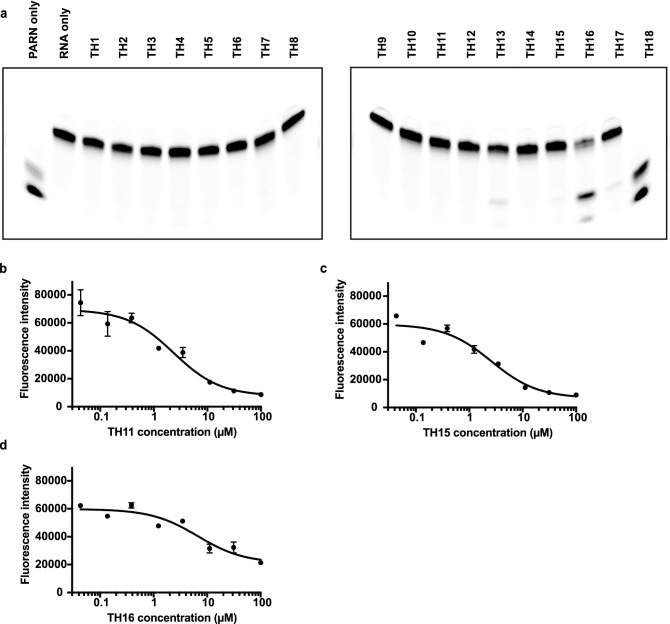
Testing high-throughput screening hits using gel assay. (**a**) Gels illustrating inhibitory effects of different drugs on PARN_1-430_. The reaction was performed the same as the fluorescence assay, heat inactivated, then loaded and visualized on gels. Full gels are presented in Supplementary Fig. S1. (**b**) Dose-response curve of TH11 on PARN_1-430_. (**c**) Dose-response curve of TH15 on PARN_1-430_. (**d**) Dose-response curve of TH16 on PARN_1-430_.

To visualize the inhibitory effects of these compounds on PARN, the reactions were run on gels. We showed all compounds could inhibit PARN at 20 μM, except for TH18 (Fig. 6). This result was consistent with the fluorescence assay, suggesting that these compounds can inhibit PARN in vitro.

### Examine inhibitory effects of identified compounds on PARN in cells

To test if the compounds identified by docking and HTS affect PARN in cells, we examined the effect of GNF-7, and the compounds from the HTS on RNAs previously known to be affected by PARN activity. Specifically, previous studies showed the levels of telomerase RNA, hTR, and several miRNAs, including miR-21-5p, decreased when PARN is depleted in Hela cells^9,11^. Therefore, we treated Hela cells with all the compounds (50 µM for GNF-7 and 10 µM for TH1-18) for 2 days and measured the levels of these RNAs using northern blotting and/or RT-qPCR.

**Figure 7:**
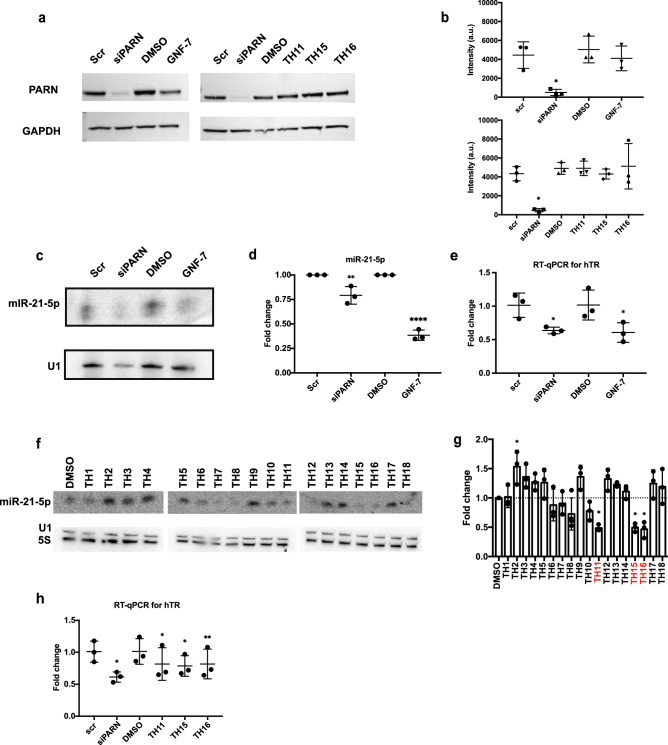
GNF-7, TH11, TH15, and TH16 inhibit PARN in cells. (**a**) Representative western blots showing GNF-7, TH11, TH15, and TH16 treatments do not affect PARN level. Hela cells were treated with siPARN and 50 μM GNF-7 (or 10 μM TH11, TH15, or TH16) (scramble siRNA and DMSO as controls) for 2 days before lysed. The blot was blotted against anti-PARN antibody. (**b**) Quantification of the changes in PARN levels of western blot using GAPDH as loading controls. siPARN and drug treatments were normalized to scramble siRNA and DMSO controls, respectively. siPARN and drug treatment data were compared to scr and DMSO data, respectively. One-way ANOVA, multiple comparisons test. Average ± SD, N = 3 biological replicates, n = 1. (**c**) Representative northern blot showing that miR-21-5p levels decreased in both PARN KD and GNF-7 treatment. Hela cells were treated with siPARN and 50 μM GNF-7 (scramble siRNA and DMSO as controls) for 2 days before RNA extraction. (**d**) Quantification of miR-21-5p levels normalized to U1 RNA. One-way ANOVA, multiple comparisons test, average ± SD, N = 3 biological replicates, n = 1. (**e**) RT-qPCR showing that hTR levels reduced in siPARN and GNF-7 treatments compared to scr and DMSO controls, respectively. One-way ANOVA, multiple comparisons test, average ± SD, N = 3 biological replicates, n = 2. (**f**) Representative northern blot showing that miR-21-5p levels decreased in TH11, TH15, and TH16 treatments. Hela cells were treated with 10 μM of TH1-TH18 (DMSO as control) for 2 days before RNA extraction. (**g**) Quantification of miR-21-5p levels normalized to U1 RNA. One-way ANOVA, multiple comparisons test, average ± SD, N = 3 biological replicates, n = 1. (**h**) RT-qPCR showing that hTR levels reduced in siPARN and drug treatments compared to scr and DMSO controls, respectively. One-way ANOVA, multiple comparisons test, average ± SD, N = 3 biological replicates, n = 2. For nothern blot and RT-qPCR quantifications, Scr and DMSO controls were independently set to 1 and used as control for siPARN and drug treatments, respectively. *P < 0.05, **P < 0.005, ***P < 0.001, ****P < 0.0001, n.s. was not indicated. Full blots are presented in Supplementary Fig. S1.

We observed that treatment with GNF-7 reduced miR-21-5p levels to ~ 35% compared to the controls when using northern blotting (Fig. 7c and d). Of the compounds from the HTS, TH11, 15, and 16 showed the strongest effects and reduced miR-21-5p levels to ~ 50% compared to the controls (Fig. 7f and g). These effects were similar to a PARN KD, which reduced miR-21-5p levels to ~ 75% compared with siRNA controls. None of the compounds reduced PARN protein levels in Hela cells (Fig. 7a and b). Moreover, RT-qPCR was done to examine the changes in hTR levels of the compounds, which confirmed a decrease in levels of hTR with compounds or siPARN treatments, compared to the controls, (Fig. 7e and h). This suggested that GNF-7, TH11, TH15, and TH16 treatments can inhibit PARN activity in cells and thereby decrease specific RNA levels.

**Figure 8:**
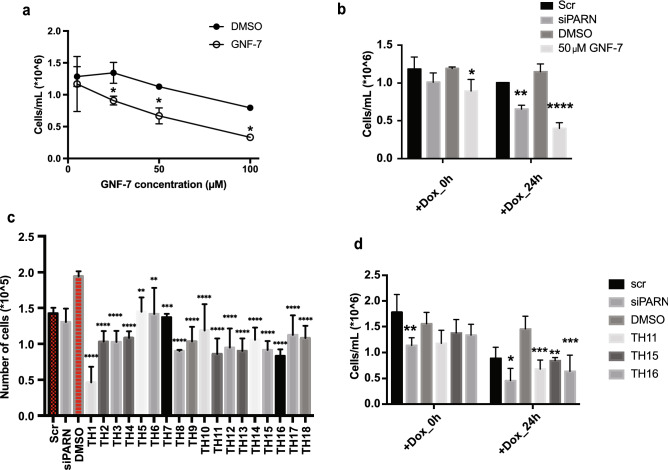
Cell death assay for GNF-7 treatment. (**a**) Numbers of cells at 2 days after DMSO and GNF-7 treatment. 2-way ANOVA, multiple comparisons test, average ± SD, N = 3 biological replicates, n = 1. (**b**) Numbers of cells at 0 and 24 h-post doxorubicin treatment. Hela cells were treated with siPARN or GNF-7 for 2 days (scramble siRNA and DMSO as controls) before adding doxorubicin. Cells were collected for quantification after 0- and 24-h post doxorubicin treatment. 2-way ANOVA, multiple comparisons test, average ± SD, N = 3 biological replicates, n = 1. *P < 0.05, **P < 0.005, ***P < 0.001, ****P < 0.0001, n.s. was not indicated. (**c**) Numbers of cells at 2 days after DMSO and TH1-TH18 treatment. One-way ANOVA, multiple comparisons test, average ± SD, N = 3 biological replicates, n = 1 (**d**) Numbers of cells at 0 and 24 h-post doxorubicin treatment. Hela cells were treated with siPARN, TH11, TH15, or TH16 for 2 days (scramble siRNA and DMSO as controls) before adding doxorubicin. Cells were collected for quantification after 0- and 24-h post doxorubicin treatment. 2-way ANOVA, multiple comparisons test, average ± SD, N = 3 biological replicates, n = 1. *P < 0.05, **P < 0.005, ***P < 0.001, ****P < 0.0001, n.s. was not indicated.

Previous work has shown that PARN inhibition leads to cell death in combination with DNA damaging agent, which has been interpreted to occur through the induction of p53^11^. Given this, we examined if GNF-7, TH11, TH15, or TH16 affected cell growth either by themselves or in combination with the chemotherapeutic agent, doxorubicin. We observed that at 25 μM GNF-7 and 10 μM of TH11, TH15, and TH16, Hela cells showed growth defects compared to DMSO treatment (Fig. 8a and c). More importantly, we observed that both PARN KD (as previously shown) and cells treated with these compounds showed increased cell death after 24 h of doxorubicin treatment compared to the scramble siRNA and DMSO controls (Fig. 8b and d). This indicates that GNF-7, TH11, TH15, and TH16 increase the sensitivity of cells to the chemotherapeutic agent, possibly through upregulating p53.

## Discussion

Herein, we report the identification of PARN inhibitors in vitro and in cells. PARN was purified and its poly(A) trimming activity was shown to be dose-dependent, which can be measured by a simple fluorescence assay (Fig. 3c). This assay is a useful tool for determining PARN enzymatic activity and for possible drug screen. In computational modeling, we identified several compounds predicted to dock with PARN and tested them using in vitro assays (Figs. 4 and 5). Moreover, with HTS, we found multiples PARN inhibitors using the fluorescence assay. These together identified GNF-7, a Bcr-Abl inhibitor, TH11, TH15, and TH16 as compounds that inhibit PARN. GNF-7, TH11, TH15, and TH16 were showed to inhibit PARN in a dose-dependent manner with a IC50 of 35 $\pm$ 13, 3.36, 2, and 7.9 μM, respectively, which are significantly lower compared to AMP (Fig. 5d, 6b–d)^31^. We also observed these compounds cause phenotypes consistent with PARN inhibition in cells with a reduction in hTR and miR-21-5p RNA levels similar to PARN KD (Fig. 7c–h).

The discovery and development of a selective and effective PARN inhibitor could be a useful tool for cancer treatment. PARN is a processive deadenylase and PARN KD has been shown to upregulate p53 protein in cancer cells, which causes cell-cycle arrest and prevents cell growth and development^11,29^. Thus, targeting PARN may offer a potential therapeutic approach for repressed p53-induced cancers. Previous reports have described aminoglycosides, synthetic nucleotides with fluoro-glucopyranosyl sugar moiety and benzoyl-modified cytosine or adenine, glucopyranosyl analogs bearing uracil, 5-fluorouracil, or thymine as base moiety, and purine-2,6-dione derivatives as possible PARN inhibitors (Table 1), although these compounds required relatively high concentrations and/or have not been shown to inhibit PARN activity in cells^30,32–34^. While some purine-2,6-dione derivatives showed PARN inhibition at relatively low concentrations: 30 μM (5b, 8a–d, and 8f), 10 μM (5j and 5k), and 3 μM (8e and 8j) using a similar fluorescence assay, only five IC50 values were reported with the lowest value of 23.9 ± 3.7 μM (compound 8j) (Table 1)^34^. The IC50 of compound 8j is slightly lower than that of GNF-7, suggesting compound 8j may be a good candidate for PARN inhibitor as well. The remainder of these previously identified PARN inhibitors were either tested with a different substrate (poly(A)) or their IC50s were not determined making a direct comparison between the effectiveness of these inhibitors on the activity of PARN difficult^30,32–34^.

From our assays, we identified the pyrimidopyrimidin-2-one GNF-7, TH11, TH15, and TH16 as PARN inhibitors. All the compounds are kinase inhibitors. GNF-7 is considered a multi-kinase inhibitor, but it is not a broad-spectrum kinase inhibitor^42^. GNF-7 is a potent inhibitor of Bcr-Abl tyrosine kinase, ACK1 (activated CDC42 kinase 1), and GCK (germinal center kinase) with IC50s of 133 nM, 25 nM, and 8 nM, respectively^41,43^. This is not unexpected since most kinase inhibitors are ATP mimetics; however, our studies support that GNF-7, TH11, TH15, and TH16 inhibit PARN activity and could be used as lead compounds for structure–activity study to develop PARN inhibitors with improved potency and selectivity.

Several observations argue GNF-7, TH11, TH15, and TH16 can inhibit PARN in cells. First, these drug treatments of cells decreased the levels of hTR and miR-21-5p (Fig. 7c–h), as is seen in PARN- deficient cells^9,11,12,17,18^. Second, we observed that GNF-7, TH11, TH15, and TH16 acted similarly to siRNA KD of PARN at increasing cell death in the presence of doxorubicin (Fig. 8b and d). Taken together, we propose that GNF-7, TH11, TH15, and TH16 can be used as chemical tools for the inhibition of PARN both in vitro and in cells. However, since these compounds can inhibit a range of kinases, finding additional PARN inhibitors, or developing derivatives of these compounds with more selectivity for PARN is an important future area of future research.

## Materials and methods

### Computational-based library docking

The SelleckChem kinase inhibitor library (SelleckChem, Cat. No. L1200) consisting of 1,820 compounds was docked into active site of the 2.6 Å human PARN nuclease domain crystal structure (PDB: 2A1R)^35^, using the Glide module within Schrödinger (Release 2020-3, Schrödinger LLC, New York, NY)^44–46^. Prior to docking, the water molecules were removed, and the proteins were prepared by assigning bond orders, adding hydrogens, and repairing any side chains or missing amino acid sequences. To complete protein preparation a restrained minimization of the protein structure was performed using the default constraint of 0.30 Å RMSD and the OPLS_2005 force field^47^. The prepared protein was subjected to SiteMap analysis^46^, that identified the catalytic binding site and docking grids were generated using Receptor Grid Generation. The compounds in the SelleckChem kinase inhibitor library were prepared using LigPrep by generating possible states at the target pH 7.0 using Epik and minimized by applying the OPLS_2005 force field^47^. Molecular docking simulations were performed using the Glide ligand docking module in XP (extra precision) mode and included post-docking minimization^45^. The docked structures were examined and high-ranked compounds with favorable XP GScores for ligand binding, that displayed interaction with residues Asp28-Phe31, the divalent metal cation binding site within the active site (Fig. 4), were selected for evaluation. The XP GScore is an empirical scoring function that approximates the ligand binding free energy; therefore, a more negative value represents favorable binding.

### High-throughput screening

2 µL of protein (100 nM final concentration) (stock protein was diluted in 1× lysis buffer (HEPES KOH, pH 7.4, 30 mM, KOAc 100 mM, and Mg(OAc)_2_ 2 mM) was added to wells using offline Multidrop Combi nL. The reaction was pre-incubated with 12.5 nL of Sygnature library (Enamine kinase library (HBL-24) (31.25 μM) for 15 min before 2 µL of RNA (10 µM) was added (DMSO was added to control wells using Echo 655). The reaction was incubated at 22 °C for 20 min in Cytomat automatic incubator. After the incubation, the reaction was quenched by adding 4 µL of quencher solution (30 µM of 3′-BHQ labeled quench DNA in 1% SDS) using Multidrop Combi nL. The quenched reaction was incubated at room temperature for 10 min and fluorescence was measured using PHERAstar FSX (λ485/520). For the counter screen, no protein was added to the reaction.

For active potency, dilution series of 10 mM of kinase library compounds (8 points 1:3 dilution, final top concentration was 100 µM) was used to generate IC50 curves. Curves were fitted within Genedata using SmartFit algorithm.

### Plasmids and Purification of recombinant PARN

Human PARN ORF was codon-optimized using iDT codon optimizer tool for bacterial expression and the corresponding gene block fragment was purchased from iDT. PcoldI-PARN plasmid with Chloramphenicol resistance containing the full-length human PARN ORF was a kind gift from Professor Yukihide Tomari^20^. Full-length PARN ORF was cut from the Pcold-PARN plasmid using NdeI-XhoI restriction digest and the native vector was gel purified. PARN 1-430 ORF fragment was PCR amplified from the gene block using the following primers and gel purified.

Fwd primer: TAAGCACATATGATGGAAATCATTCGCTCC

Rev primer: TGCTTACTCGAGTTAAATGTCCATCACACGCA

The purified PCR product was ligated to the PcoldI NdeI-XhoI digest vector using T4 DNA ligase I (NEB M0202S) and correct insertion was verified using Sanger sequencing. PARN D28A F31A double mutant was created by site-directed mutagenesis of the PARN_1-430_ expressing plasmid using the following primers and mutation insertion was verified using sanger sequencing.

Fwd primer: TTTTTTGCAATTGCAGGGGAGGCTTCCGGTATTTCC

Rev primer: GGAAATACCGGAAGCCTCCCCTGCAATTGCAAAAAA

For recombinant protein purification, the vector was expressed in Rosetta 2 DE at 37 °C overnight with Amp-Camp (50 µg/mL). The starter culture was transferred into 1 L of TB culture and incubated at 37 °C to reach O.D. of 1. The proteins were induced with 1 mM IPTG for 2 days at 15 °C. The proteins were purified using Ni-NTA column and buffer exchanged into storage buffer (30 mM Hepes KOH, pH 7.4, 100 mM KOAc, 2 mM Mg(OAc)_2_, 30% glycerol, 1 mM TCEP). Proteins were verified on SDS gels and stored at − 80 °C.

### siRNAs

siRNAs targeting PARN (siGenome) was purchased from Dharmacon in the Smartpool formulation (M-011348-00-0005). All-stars negative control siRNA from QIAGEN (SI03650318) was used as negative control.

### Cell culture

HeLa cells were purchased from ATCC (CCL2) and verified for absence of mycoplasma contamination. HeLa cells were cultured in DMEM containing 10% FBS, 1% Pen/Strep, at 37 °C under ambient conditions.

HeLa cells were seeded ~ 100,000 cells/well in a six-well plate 24 h before transfection/GNF-7 (50 µM) treatment. siRNA transfection was performed using Lipofectamin RNAiMAX (Thermo Fisher Scientific) as per manufacturer’s protocol. 48 h after transfection/drug treatment, cells were collected for either RNA or protein analysis.

### RNA extraction and Northern blotting

Total RNA was extracted from cell lysates using TriZol as per manufacturer’s protocol and DNAse treated. After quantification on Nanodrop, total RNA was separated on an acrylamide 7 M Urea gel. RNA was transferred to a nylon membrane (Nytran SPC, GE Healthcare) using semiwet transfer. After UV/EDC crosslinking, the blot was pre-hybridized and hybridized in PerfectHyb Plus Hybridization Buffer (Sigma Aldrich) at 42 °C. Northern probes have been previously described^11,48^. After hybridization and washing in 2 × SSC 0.1% SDS wash buffer, blots were exposed to a cassette and imaged on a Typhoon FLA 9500 Phosphoimager. Band intensities were quantified using ImageJ and normalized to the U1 levels under each condition.

### RT-qPCR

Extracted total RNA was reverse transcribed using Mir-X miRNA first strand synthesis kit (Taraka, Cat # 638315) to make cDNA and qPCR was perfomred with iQ SYBR Green Supermix (BioRad, Cat. No. 1708880) with CGCTGTTTTTCTCGCTGACT (forward primer) and GCTCTAGAATGAACGGTGGAA (reverse primer) for hTR. The RNA levels were normalized using 5S rRNA as a housekeeping gene.

### Western blotting

Cells was lysed with 2× lysis buffer (2.5% SDS, 4% BME, protease inhibitor) and was separated on a 4–12% Bis–Tris NuPage gel (ThermoFisher) and transferred to protran membrane (Amer- sham). After blocking in 5% non-fat milk in 1×TBST, blots were probed with anti-PARN (Abcam, ab188333, 1:1000 dilution) overnight at 4 °C and HRP anti-rabbit goat (Cell Signaling Technology, 7074S, 1:1000 dilution) secondary antibody for one hour. Blot was quantified using ImageJ and normalized to GAPDH levels (GAPDH antibody (0411) HRP) (Santa Cruz Biotechnology, sc-47724 HRP).

### Inhibition fluorescence assay

1 µL of protein (73 nM as final concentration) (stock protein is diluted in 1× lysis buffer (HEPES KOH, pH 7.4, 30 mM, KOAc 100 mM, and Mg(Oac)_2_ 2 mM) was added to 4 µL of 2.5× reaction buffer (Tris–HCl pH 7.4, 10 mM, KCl 50 mM, MgCl_2_ 5 mM). If drug was added, reaction was pre-incubated with drugs for 10 min before 5 µL of RNA (5 µM as final concentration) was added. The reaction was incubated at 37 °C for 20 min. After the incubation, the reaction was either diluted with 2× loading buffer and heated to 95 °C for 5 min for gel or quenched by adding 10 µL of quencher solution (30 µM of 3′-BHQ labeled quench DNA in 1% SDS). Quenched reaction was incubated at room temperature for 10–60 min and fluorescence was measured using Fluorescein wavelength measurement.

### Gels

15% TBE-Urea gel (Thermo Fisher Scientific) was prerun at 20W for 30 min. RNAs from the reaction was loaded into 15% TBE gels and run at 300 V for 35 min. The gel was visualized using iBright (Invitrogen FL1500).

### Cell death assay

The same number of Hela cells were seed into 6 well-plates and treated with GNF-7 (SelleckChem, S8140), TH1-18, or transfected for 2 days. Cell counting were done 2 days post-treatment. For doxorubicin (EMD Millipore, 504042) treatment, doxorubicin (1 μM) was added to cells. Cells were collected and counted at 0 h and 24 h after treatment.

## Supplementary Information

Supplementary Figures.

## Data Availability

The datasets generated during and/or during the current study are available from the corresponding author on reasonable request.

## References

[1] Dehlin E, Wormington M, Körner CG, Wahle E (2000). Cap-dependent deadenylation of mRNA. EMBO J..

[2] Gao M, Fritz DT, Ford LP, Wilusz J (2000). Interaction between a poly(A)-specific ribonuclease and the 5' cap influences mRNA deadenylation rates in vitro. Mol. Cell.

[3] Körner CG, Wahle E (1997). Poly(A) tail shortening by a mammalian poly(A)-specific 3'-exoribonuclease. J. Biol. Chem..

[4] Martînez J, Ren YG, Nilsson P, Ehrenberg M, Virtanen A (2001). The mRNA cap structure stimulates rate of poly(A) removal and amplifies processivity of degradation. J. Biol. Chem..

[5] Copeland PR, Wormington M (2001). The mechanism and regulation of deadenylation: Identification and characterization of Xenopus PARN. RNA.

[6] Kim JH, Richter JD (2006). Opposing polymerase-deadenylase activities regulate cytoplasmic polyadenylation. Mol. Cell.

[7] Körner CG (1998). The deadenylating nuclease (DAN) is involved in poly(A) tail removal during the meiotic maturation of Xenopus oocytes. EMBO J..

[8] Berndt H (2012). Maturation of mammalian H/ACA box snoRNAs: PAPD5-dependent adenylation and PARN-dependent trimming. RNA.

[9] Shukla S, Schmidt JC, Goldfarb KC, Cech TR, Parker R (2016). Inhibition of telomerase RNA decay rescues telomerase deficiency caused by dyskerin or PARN defects. Nat. Struct. Mol. Biol..

[10] Shukla S, Parker R (2017). PARN modulates Y RNA stability and its 3'-end formation. Mol. Cell Biol..

[11] Shukla S, Bjerke GA, Muhlrad D, Yi R, Parker R (2019). The RNase PARN controls the levels of specific miRNAs that contribute to p53 regulation. Mol. Cell.

[12] Roake CM (2019). Disruption of telomerase RNA maturation kinetics precipitates disease. Mol. Cell.

[13] Ustianenko D (2016). TUT-DIS3L2 is a mammalian surveillance pathway for aberrant structured non-coding RNAs. EMBO J..

[14] Chang HM, Triboulet R, Thornton JE, Gregory RI (2013). A role for the Perlman syndrome exonuclease Dis3l2 in the Lin28-let-7 pathway. Nature.

[15] Heo I (2008). Lin28 mediates the terminal uridylation of let-7 precursor MicroRNA. Mol. Cell.

[16] Lardelli RM, Lykke-Andersen J (2020). Competition between maturation and degradation drives human snRNA 3' end quality control. Genes Dev..

[17] Tseng CK (2015). Human telomerase RNA processing and quality control. Cell Rep..

[18] Moon DH (2015). Poly(A)-specific ribonuclease (PARN) mediates 3'-end maturation of the telomerase RNA component. Nat. Genet..

[19] Son A, Park JE, Kim VN (2018). PARN and TOE1 constitute a 3' end maturation module for nuclear non-coding RNAs. Cell Rep..

[20] Katoh T, Hojo H, Suzuki T (2015). Destabilization of microRNAs in human cells by 3' deadenylation mediated by PARN and CUGBP1. Nucleic Acids Res..

[21] Agarwal V, Bell GW, Nam JW, Bartel DP (2015). Predicting effective microRNA target sites in mammalian mRNAs. Elife.

[22] Liu J, Zhang C, Zhao Y, Feng Z (2017). MicroRNA control of p53. J. Cell Biochem..

[23] Swarbrick A (2010). miR-380-5p represses p53 to control cellular survival and is associated with poor outcome in MYCN-amplified neuroblastoma. Nat. Med..

[24] Vlachos IS (2015). DIANA-miRPath v3.0: Deciphering microRNA function with experimental support. Nucleic Acids Res..

[25] Burns DM, D'Ambrogio A, Nottrott S, Richter JD (2011). CPEB and two poly(A) polymerases control miR-122 stability and p53 mRNA translation. Nature.

[26] Rivlin N, Brosh R, Oren M, Rotter V (2011). Mutations in the p53 tumor suppressor gene: Important milestones at the various steps of tumorigenesis. Genes Cancer.

[27] Bieging KT, Mello SS, Attardi LD (2014). Unravelling mechanisms of p53-mediated tumour suppression. Nat. Rev. Cancer.

[28] Joerger AC, Fersht AR (2016). The p53 pathway: Origins, inactivation in cancer, and emerging therapeutic approaches. Annu. Rev. Biochem..

[29] Zhang LN, Yan YB (1853). Depletion of poly(A)-specific ribonuclease (PARN) inhibits proliferation of human gastric cancer cells by blocking cell cycle progression. Biochim. Biophys. Acta.

[30] Balatsos NA (2009). Competitive inhibition of human poly(A)-specific ribonuclease (PARN) by synthetic fluoro-pyranosyl nucleosides. Biochemistry.

[31] Balatsos NA, Anastasakis D, Stathopoulos C (2009). Inhibition of human poly(A)-specific ribonuclease (PARN) by purine nucleotides: Kinetic analysis. J. Enzyme Inhib. Med. Chem..

[32] Balatsos N (2012). Kinetic and in silico analysis of the slow-binding inhibition of human poly(A)-specific ribonuclease (PARN) by novel nucleoside analogues. Biochimie.

[33] Ren YG, Martínez J, Kirsebom LA, Virtanen A (2002). Inhibition of Klenow DNA polymerase and poly(A)-specific ribonuclease by aminoglycosides. RNA.

[34] Jadhav GP (2015). Discovery, synthesis and biochemical profiling of purine-2,6-dione derivatives as inhibitors of the human poly(A)-selective ribonuclease Caf1. Bioorg. Med. Chem. Lett..

[35] Wu M (2005). Structural insight into poly(A) binding and catalytic mechanism of human PARN. EMBO J..

[36] Martinez J (2000). A 54-kDa fragment of the Poly(A)-specific ribonuclease is an oligomeric, processive, and cap-interacting Poly(A)-specific 3' exonuclease. J. Biol. Chem..

[37] Maryati M (2014). A fluorescence-based assay suitable for quantitative analysis of deadenylase enzyme activity. Nucleic Acids Res..

[38] Ren YG, Martínez J, Virtanen A (2002). Identification of the active site of poly(A)-specific ribonuclease by site-directed mutagenesis and Fe(2+)-mediated cleavage. J. Biol. Chem..

[39] Ren YG, Kirsebom LA, Virtanen A (2004). Coordination of divalent metal ions in the active site of poly(A)-specific ribonuclease. J. Biol. Chem..

[40] Lai WS, Kennington EA, Blackshear PJ (2003). Tristetraprolin and its family members can promote the cell-free deadenylation of AU-rich element-containing mRNAs by poly(A) ribonuclease. Mol. Cell Biol..

[41] Qin X (2020). The Bcr-Abl inhibitor GNF-7 inhibits necroptosis and ameliorates acute kidney injury by targeting RIPK1 and RIPK3 kinases. Biochem. Pharmacol..

[42] Liang X (2016). Discovery of 2-((3-Amino-4-methylphenyl)amino)-N-(2-methyl-5-(3-(trifluoromethyl)benzamido)phenyl)-4-(methylamino)pyrimidine-5-carboxamide (CHMFL-ABL-053) as a potent, selective, and orally available BCR-ABL/SRC/p38 kinase inhibitor for chronic myeloid leukemia. J. Med. Chem..

[43] Nonami A (2015). Identification of novel therapeutic targets in acute leukemias with NRAS mutations using a pharmacologic approach. Blood.

[44] Friesner RA (2004). Glide: A new approach for rapid, accurate docking and scoring. 1. Method and assessment of docking accuracy. J. Med. Chem..

[45] Friesner RA (2006). Extra precision glide: Docking and scoring incorporating a model of hydrophobic enclosure for protein-ligand complexes. J. Med. Chem..

[46] Halgren TA (2009). Identifying and characterizing binding sites and assessing druggability. J. Chem. Inf. Model..

[47] Beckstein O, Fourrier A, Iorga BI (2014). Prediction of hydration free energies for the SAMPL4 diverse set of compounds using molecular dynamics simulations with the OPLS-AA force field. J. Comput. Aided Mol. Des..

[48] Xi L, Cech TR (2014). Inventory of telomerase components in human cells reveals multiple subpopulations of hTR and hTERT. Nucleic Acids Res..

