# Supplementary material for: Identification of PARN nuclease activity inhibitors by computational-based docking and high-throughput screening
Source: Sci Rep. 2023 Mar 31;13:5244. doi: 10.1038/s41598-023-32039-z (PMC10066322; doi:10.1038/s41598-023-32039-z)
Supplement: Supplementary file 1 — Supplementary Figures. [file 41598_2023_32039_MOESM1_ESM.pdf]

Figure S1. Original images of gels and blots acquired during the study.

Uncropped Figure 3c

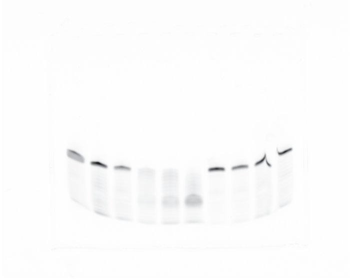

Uncropped Figure 3d

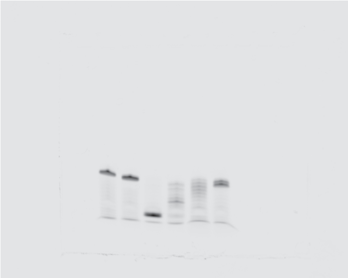

Uncropped Figure 5b (top)

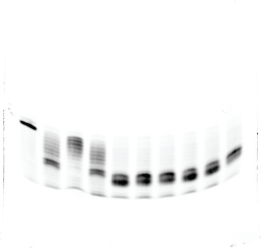

Uncropped Figure 5b (bottom)

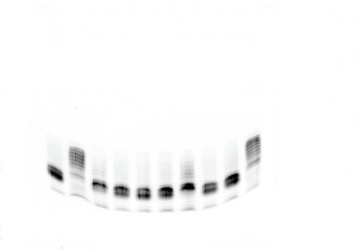

Uncropped Figure 7a (PARN)

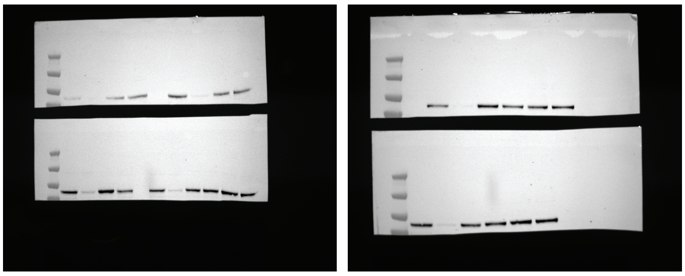

Uncropped Figure 7a (GAPDH)

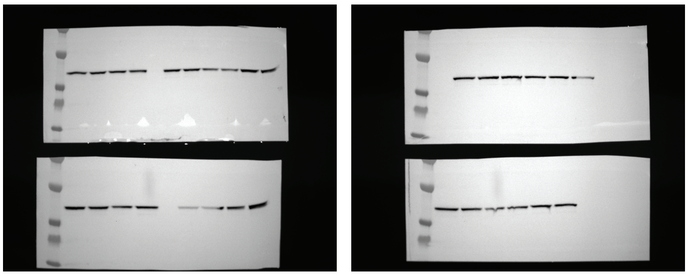

Uncropped Figure 7c (miR-21-5p)

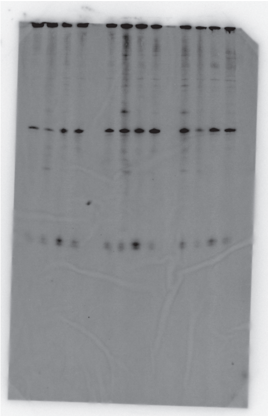

Uncropped Figure 7c (U1+5S)

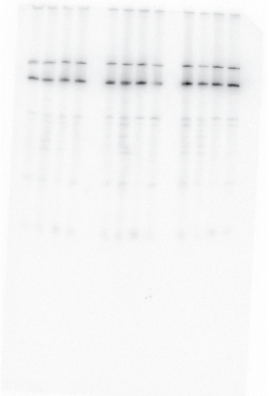

Uncropped Figure 7h (miR-21-5p)

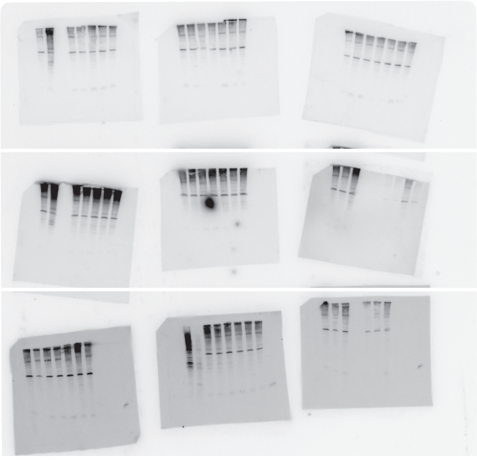

Uncropped Figure 7h (U1+5S)

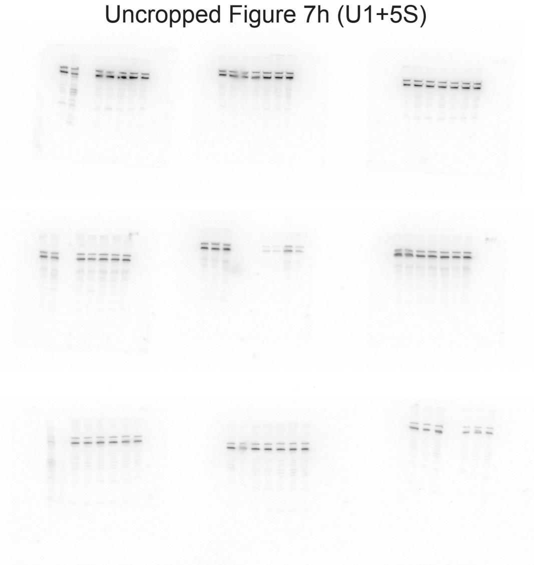

Figure S2. Chemical structures of TH11, TH15, and TH16

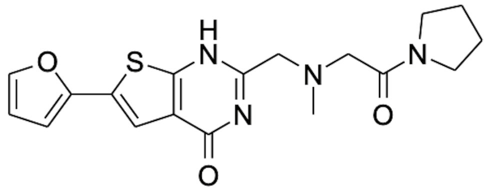

TH11

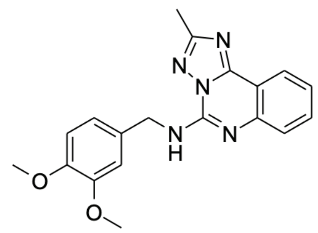

TH15

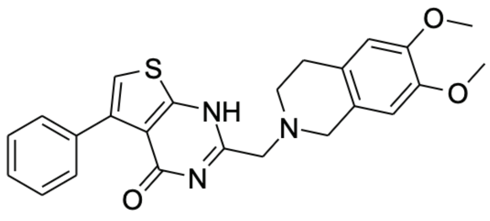

TH16
